# Experimental Researches on the Functions of the Ganglionic Nerves

**Published:** 1831-01-01

**Authors:** 


### Recherches Experimentales sur les Fonctions du Systeme Nerveux Ganglionnaire, &c.—That is:—Experimental Researches respecting the Functions of the Ganglionic System of Nerves, and their Application to Pathology. By *M. Brachet*, M.D. Octavo, Paris, 1830.

The work itself not having reached us, we are indebted to a very able analysis of it, drawn up by Dr. Sandras of Paris, for the present notice; and we conceive our readers will not regret the perusal of this article, the subject being very interesting. We may glance at our actual knowledge of the functions of the ganglionic system, prior to the publication of Dr. Brachet's work.

Willis regarded this system as a nerve placed between the perceptive function of the brain and the involuntary actions of the viscera. Vieussen's opinion was nearly similar. Lancisi regarded the ganglia as having a function somewhat analogous to that of the heart—namely, that they accelerated the movements of the nervous fluid. According to Winslow, the ganglia are so many little brains, or separate origins of the grand sympathetic nerve. Meckel believed that they produce the division of the rami into ramusculi, or filaments, and regulate the destination of the latter—and also that they unite several rami into one fasciculus. Zin partakes of the same opinion, and adds that the different rami are completely confounded in the ganglia, and never go out in juxta-position, as from the plexus. Scarpa coincides with Meckel and Zin, and traces their origin to the spinal marrow and to the fifth and sixth pairs. Bichat illustrated the views of Winslow, and referred the ganglionic system to the "organic life." Reil remarks that the great sympathetic nerve exists alone in some classes of animals, while in others of a superior nature, there is added the cerebral system of nerves. He maintains that every part of the ganglionic system is originally formed in the spot where we find it, which is not the case with the cerebral system. He sees the ganglionic nerves accompany and envelop all the vessels—send conductors to the cerebral system, and thus put into communication with each other the two otherwise isolated systems of nerves. Thus voluntary life belongs to the brain—involuntary to the ganglia system. This last is devoid of common sensation in health; but when deranged, its character changes, and it becomes the conductor of sensations. "Animal magnetism (says Reil) establishes also a communication of sensations between these two spheres. Numerous examples prove that people have seen through the epigastrium." Wilson Philip believes that the ganglia serve as reservoirs for the concentration of the nervous power, flowing from the brain and spinal marrow. The views of Broussais are difficult to understand, and we pass them over. Lobstein has written an elaborate work, "De Nervi Sympathetici Fabrica et Usu." He observes that we perceive a ganglionic system in the invertebrated animals—that, in the lowest ranges of these, we see only a nervous mass serving the purpose of nutrition, whilst in others, a little more elevated in the scale, additional masses are appended:—In animals still higher, the nervous matter of organs superintending motion and feeling, is concentrated in one mass, the brain—where ganglia disappear;—and where, on the contrary, there is a ganglionic system appropriated solely to the organs of nutrition.

"No doubt (says Lobstein) the grand sympathetic, like the nerves in general, is the source of the vital principle, in the parts to which it belongs—preserving their tone, and distributing to them energy and action. This nerve presides over nutrition, secretion, and the circulation—is the link of union between the intellectual and organic functions, &c."

But it is time to proceed to the work of M. Brachet himself.

### SECT. I.—GENERAL FUNCTIONS.

There is no function without agents, without instruments. There is no bile without a liver—no sensations without nerves. All organs feel—and therefore all organs have nerves. But vegetables also have sensibility. They have the functions of absorption, nutrition, exhalation, circulation, generation, and destruction—they only want *digestion* to bring them pretty nearly on a par with animals. All these last have a ganglionic system—and vegetables, M. Brachet thinks, have their's—the medulla or pith. The various experiments and arguments in proof of this we must pass over. According to this author, there are no such things as vital powers or properties—"il n'y a point de propriétés vitales"—these are the results of the actions of organs, in a word, their functions—and he thinks it would be true physiology to admit, instead of these assumed vital powers—1mo, perceptible sensation (through the medium of the cerebral nerves)—2ndo, non-perceptible sensations, (through the ganglionic nerves)—3tio, muscular contraction, *voluntary* in the loco-motive muscles—*involuntary* in the muscular fibres of the viscera—4to, capillary contraction.

He would wish, to discard all ONTOLOGY in physiology as in medicine—and that we should only see in the healthy living body, tissues, organs, and fluids—in diseases, only an alteration in these principles.

### SECT. II.—SPECIAL FUNCTIONS OF THE GANGLIONIC SYSTEM.

M. Brachet only adverts to the principal functions of this system—first, as to the heart—2d, as to the respiration—3d, as to the stomach—4th, as to the intestines—5th, as to the bladder—6th, the organs of generation—7th, the secretions—8th, the sympathies—9th, vision—10th, various passions.

I. THE HEART. This important organ receives a number of nervous filaments from the great sympathetic—a few from the pneumo-gastric. On removing the cerebrum and cerebellum, the motions of the heart are not arrested. If death takes place suddenly when we remove the origin of the pneumo-gastric nerves, it is by asphyxia, and life may be prolonged for some time by artificial respiration. From experiments and reasoning M. Brachet comes to the conclusion, contrary to Legallois, that the spinal marrow itself does not govern the motions of the heart. He has pushed a wire *slowly* through the spinal marrow of animals, and has not affected the heart's action till the instrument reached the cauda equina. Anatomical researches have proved, he thinks, that the brain and spinal marrow are formed posterior to the heart, and consequently after the motion of that organ has commenced. Fœtuses have lived seven or eight months without spinal marrow. Numerous experiments have shewn that the heart's action goes on for days after section of the pneumo-gastric nerves. The trouble of function in the heart, consequent on these operations, results from the consent between the two systems of nerves. That the heart's action is kept up by the grand sympathetic is proved, in Dr. B's opinion, by several experiments—among others, the following. He divided all the filaments going from the internal cervical ganglia to the cardiac plexus. Sometimes the action of the heart ceased completely—sometimes it ceased for a moment, and then re-commenced. Surprised at this diversity of result, M. Brachet knew not what to think; but reflecting that the ganglia were all isolated, and, as it were, independent organs, he concluded that he must attack the cardiac ganglion itself. This he did. The operation is difficult, but whenever he was able to remove the ganglion entirely, the action of the heart ceased instantly. When it was only partially removed, the action of the heart went on for some time. The nervous system, then, of the heart commences with the cardiac ganglion, as was observed by Ackermann and Malpighi.

II. Influence of the Ganglionic System on the Lungs. On section of the par vagum, animals die tranquilly, and without any efforts to breathe, when immersed in water, in a vacuum, in azotic gas, which is not the case with those animals who are so immersed, but with the said nerves undivided. On destroying the origin of the eighth pair, the respiration is at once suspended, although a canula is kept in the trachea. Acephali and anacephali that have breathed after death, have the eighth pair of nerves —those who do not breathe after coming into the world, do not possess the said nerves. The author, however, relates an experiment where section of the pneumo-gastric nerves, without tracheotomy, was followed by violent efforts to breathe. He accounts for this contradictory phenomenon, by supposing that the *habit* of the respiratory muscles is continued for some time after the nervous power is cut off. By various experiments, he comes to the conclusion that, after section of the eighth pair, animals die from the accumulation of mucus in the bronchial tubes, from inability to expectorate it. The blood cannot then be oxigenated, and the animal succumbs from engorgement of the lungs. The muscles of the chest are, of course, under the influence of the cerebro-spinal system; but sanguification appears to be in the domain of the ganglionic system.

III. Influence of the Ganglionic System on the Functions of the Stomach. This organ receives nerves from the ganglionic system, and also from the eighth pair. Its office is to warn us of hunger—to produce muscular movements—and to effect chymification. If an animal is kept fasting till great sense of hunger is induced, and the par vagum be then cut, the sense of hunger ceases. They will eat till the œsophagus is filled. It is by means of the par vagum that impressions are conveyed from the stomach to the brain—a fact easily proved by section of these nerves, which obliterates all the phenomena resulting from mutual influence of stomach on brain, and brain on stomach. The suspension of digestion by section of the pneumo-gastric nerves is well known in this country, as well as the apparent re-establishment of that process by means of galvanism.

It has been ascertained that secretion of the gastric juice continues after section of the pneumo-gastric nerves, as also absorption in the stomach. These two functions, then, are concluded to be under the dominion of the ganglionic system.

IV. ON THE SMALL INTESTINES. What applies to the stomach will pretty nearly apply to the small intestines, excepting the sense of hunger. Section of the pneumo-gastric nerves has been found to paralyze the duodenum, and the upper portion of the small intestines. The inferior portion is under the influence of spinal marrow. An experiment demonstrated this unequivocally. The section of the par vagum does not stop exhalation, secretion, or absorption, in the above portion of the intestinal canal. These functions then, as in the stomach, are under the guidance of the ganglionic nerves. The lower portion of the ileum, the colon, and the rectum, as well as the bladder, are governed by the cerebro-spinal system, as far as motion and the sense of nisus to evacuate are concerned.

V. GENITAL ORGANS. The spermatic secretion, like all the other secretions, is under the influence of the ganglionic nerves. The hinder extremities of animals were paralyzed by section of the spinal marrow, but spermatic secretion went on. Conception took place in animals whose spinal marrow was divided. In parturition, however, section of the spinal marrow paralyses those muscles which assist the expulsion of the fœtus. Galvanism set these muscles into action. A female who was paraplegiac, was obliged to be delivered by the forceps. All the secretions, as was before observed, and as M. Brachet has proved by numerous experiments, are under the influence of the ganglionic system. Section of the spinal marrow does not arrest their progress.

VI. THE SYMPATHIES. The author has made some curious experiments on the influence of the cerebro-spinal and ganglionic nerves on the sympathies. He cut and tied the œsophagus of a dog, after being fed, and on irritating the uvula, efforts to vomit took place. Hence he concludes that sympathy is not merely from continuity of structure. By cutting the pneumo-gastric nerves the foregoing sympathy was annihilated. The trachea was divided, as also the inferior portion of the cervical medulla, and sneezing was capable of being provoked, though there was no other communication between the nose, to which irritants were applied, and the lungs, except the par vagum. The par vagum being divided, no sneezing could be produced.

The cerebro-spinal nerves, then, are media through which some sympathies are effected. But are they the sole media ? No. As the ganglionic nerves cannot excite those actions which are under the influence of the cerebro-spinal system, so the nerves of this last system cannot act directly on functions superintended by the ganglionic nerves. Numerous experiments and proofs are offered that the ganglionic nerves are devoid of cerebral sensibility in a state of health, but possessed of it exquisitely when irritated. They are proved to lose this new kind of sensibility when the spinal branches of the ganglia have been cut—hence it is inferred that it is by means of the spinal nerve solely that these ganglia become *cerebrally* sensible, when in a state of irritation. This *cerebral sensibility* never exists in the ganglionic nerves, except in the very point which is irritated :—hence our author concludes that this extra-sensibility is *not generated* in these nerves, but that they transmit to their nervous centre the impression which they receive, and the cerebral nerves draw it up from thence. Thus, in irritations of the ganglionic nerves, the pain is not felt in the part irritated, but in the ganglion to which the nerve goes—hence the pains felt in phthisis, hepatitis, &c. In fine, the pain is local (and truly indicated as to seat) when the encephalic nerves are more affected in one part than in another—while it is always *at some distance* from the seat of irritation, when the ganglionic nerves are affected. This last system, then, is capable of transmitting sympathies—but only in its own manner. Thus we have cerebral sympathies, ganglionic sympathies—and mixed sympathies, where irritations in the ganglionic system are communicated to the cerebro-spinal. The author applies his doctrine to several organs—to the brain, for instance, shewing how it sympathises with the organs of sense, the heart, lungs, and digestive viscera, &c.

VII. Influence of the Ganglionic Nerves on Vision. From numerous experiments our author concludes that the regular movements of the iris are under the influence of the ophthalmic ganglion, and that the impression of light does not act on the pupil but through the intervention of the mixed sympathy above alluded to. He has destroyed the ophthalmic ganglion—the iris remained immoveable—all the other functions of the eye remaining unaffected. The other eye of the animal was destroyed, but vision remained unimpaired. The conclusion is, that the *cerebral* nervous system receives the luminous impressions—that the ganglionic nerves preside over the motions of the iris—and that the sympathy which exists between the iris and the retina is of a cerebro-ganglionic nature. By section of the cervical filament of the great sympathetic, the eye became lachrymose and painful—the conjunctiva red and thickened, without pain and by atony.

The last chapter of the work is on the influence of the ganglionic system on the passions. Dr. Brachet admits that the passions are developed by means of impressions on the sensorium through the medium of the senses and the imagination, as proved by love, anger, hatred, joy, sorrow, &c. But he truly remarks that the ganglionic system is not inert in these cases. The connexion which subsists between the intellectual and automatic life enables the latter to modify our passions to an extent much greater than is generally imagined. The reviewer, however, has not analyzed this part of the work, and we are forced to take his word for the ability with which the inquiry is conducted by M. Brachet. When possessed of the original, we shall probably offer some more minute details of particular portions of the Essay—contenting ourselves, at present, with the foregoing analysis, as given by a very able continental cotemporary—the Journal de Medecine Pratique.

